# Selectivity of Mixed Iron-Cobalt Spinels Deposited on a N,S-Doped Mesoporous Carbon Support in the Oxygen Reduction Reaction in Alkaline Media

**DOI:** 10.3390/ma14040820

**Published:** 2021-02-09

**Authors:** Aldona Kostuch, Joanna Gryboś, Szymon Wierzbicki, Zbigniew Sojka, Krzysztof Kruczała

**Affiliations:** Faculty of Chemistry, Jagiellonian University in Krakow, Gronostajowa 2, 30-387 Krakow, Poland; kostuch@chemia.uj.edu.pl (A.K.); grybosjo@chemia.uj.edu.pl (J.G.); szymon.wierzbicki@student.uj.edu.pl (S.W.)

**Keywords:** oxygen reduction reaction (ORR), spinel iron-cobalt oxides, doped mesoporous carbon support, electrocatalysts, metal-air batteries, fuel cells

## Abstract

One of the practical efforts in the development of oxygen reduction reaction (ORR) catalysts applicable to fuel cells and metal-air batteries is focused on reducing the cost of the catalysts production. Herein, we have examined the ORR performance of cheap, non-noble metal based catalysts comprised of nanosized mixed Fe-Co spinels deposited on N,S-doped mesoporous carbon support (N,S-MPC). The effect of the chemical and phase composition of the active phase on the selectivity of catalysts in the ORR process in alkaline media was elucidated by changing the iron content. The synthesized materials were thoroughly characterized by transmission electron microscopy (TEM), X-ray diffraction (XRD), and Raman spectroscopy (RS). Detailed S/TEM/EDX and Raman analysis of the phase composition of the synthesized ORR catalysts revealed that the dominant mixed iron-cobalt spinel is accompanied by minor fractions of bare cobalt and highly dispersed spurious iron oxides (Fe_2_O_3_ and Fe_3_O_4_). The contribution of individual phases and their degree of agglomeration on the carbon support directly influence the selectivity of the obtained catalysts. It was found that the mixed iron-cobalt spinel single phase gives rise to significant improvement of the catalyst selectivity towards the desired 4e^−^ reaction pathway, in comparison to the reference bare cobalt spinel, whereas spurious iron oxides play a negative role for the catalyst selectivity.

## 1. Introduction

Design and fabrication of new electrocatalysts for oxygen reduction reaction (ORR) with high activity, selectivity, and durability is one of the most important challenges in the context of practical use of the rechargeable metal-air batteries as well as the anion-exchange membrane (AEMFCs) and microbial fuel cells [1,2,3,4,5,6,7]. Development of those green and sustainable technologies is motivated by the growing demand for alternative energy sources with low environmental impact [8,9,10]. AEMFC devices are attracting increasing attention primarily due to their potential to considerably reduce the cost of fuel cell manufacture and operation [11,12]. The main impediments on the progress in this subject are the high cost and scarcity of the most efficient platinum-based ORR catalysts, which has stimulated researchers to look for cheaper alternative materials, which performance is equivalent to the Pt benchmark. Transition metal oxides of the spinel structure due to their unique redox properties and facile control of their composition and morphology are considered as an interesting group of catalytic materials for many photo/electro/catalytic reactions, including the ORR in the alkaline conditions [13,14,15,16]. Moreover, owing to their good activity, low cost, simple preparation, and high stability they are extensively investigated as electrocatalysts [17]. Among spinel-type oxides, cobalt spinel exhibits very good catalytic properties for ORR in alkaline media [3,18,19]. However, a rather small natural abundance, relatively high costs on the market, and cobalt toxicity generate incentives to reduce its content in the catalysts dedicated to practical applications. Substitution of Co by more abundant transition metals such as manganese or iron might lead to the development of cheaper and environmentally more benign catalysts. Indeed, intensively investigated Mn-Co spinels deposited on carbon carriers exhibit very good electrocatalytic properties [13,20,21,22]. Similarly, Fe-Co spinels are also recognized as promising ORR catalytic materials for metal-air batteries and AEM fuel cells [23,24,25,26,27,28]. The possibility of structural modification of these materials, by reversing their crystallographic structure from normal to inverse spinels, controlled simply by the Fe content in the sample creates one of the possibilities of improving their electroactivity. DFT calculations have revealed that an increase in the intrinsic activity in the ORR could be related to the simultaneous presence of both the Fe and Co cations in the octahedral 16d positions [23,29]. Besides the effect of the type of the spinel structure, tuning of the chemical composition of mixed spinels may allow for the generation of electrocatalytic active sites with tailored redox properties to obtain materials with the optimal catalytic performance [30,31]. Due to the semiconductor nature of spinels, carbon materials are commonly used as supports for this kind of electrocatalysts [13,14,15,25]. Not only owing to their high electrical conductivity, but also because of high specific surface area, porosity, and synergistic coupling between the carbon support and the oxide phase, which can play a key role in increasing the performance of the resultant catalysts, in terms of both their ORR activity, selectivity and durability [32,33]. As a result, a great deal of research has been devoted to the development of effective modification methods of the carbon carriers in order to improve the interfacial contact and interaction between the spinel active phase and the support [34,35,36]. Doping of carbon materials by various heteroatoms, such as sulfur, nitrogen, phosphorus, and boron have been reported as promising approaches to increase the surface polarity, regulate the acid-base properties, and create active adsorption and/or catalytic reaction centers [26,37,38,39]. 

In the present studies, we examined the selectivity of electrocatalysts obtained by partial substitution of Co cations in a cobalt spinel active phase by the cheap and eco-friendly element, such as iron, with simultaneous use of carbon support promoted with nitrogen and sulfur. The role of the Fe-Co oxide composition in controlling the favorable formation of the mixed spinel active phase, competing with undesired segregation iron oxides was also elucidated in the context of the catalyst selectivity. 

## 2. Materials and Methods

### 2.1. Materials

#### 2.1.1. Synthesis of Nitrogen and Sulfur Doped Mesoporous Carbon Support (N,S-MPC)

To obtain mesoporous carbon replica materials (MPC), acting as a support for the prepared mixed spinel catalysts, the nanoreplication method with the MCM-48 mesoporous silica serving as a template was applied [40]. Sucrose as a carbon precursor was deposited in the pores of the MCM-48 silica sieve, in the presence of sulfuric acid, being the catalyst, via the incipient wetness impregnation method. The impregnation procedure and subsequent preliminary heating were repeated twice. For 1 g of MCM-48 template two impregnation solutions consisting of (i) 4.98 g of water, 1.25 g of sucrose, and 0.14 g of sulfuric acid and (ii) 2.98 g of water, 0.75 g of sucrose, and 0.08 g of sulfuric acid were used. After each deposition, thermal pre-treatment of the produced composite was carried out at 100 °C overnight, and then at 160 °C for 6 h. In the next step, the obtained material was carbonized in a tube furnace (Czylok, Jastrzębie Zdrój, Poland) at 900 °C for 2.5 h (at a heating rate of 5 °C min^−1^), in a flow of Ar (40 cm^3^ min^−1^). To introduce heteroatoms (N and S), the prepared C/MCM-48 composite was stirred in an ethanolic thiourea solution (1 g C/MCM-48/0.7 g thiourea) for 5 h at room temperature. Then, the mixture was dried at 80 °C, overnight. Then, the sample was heat-treated at 950 °C for 30 min (with a heating rate of 10 °C min^−1^), under Ar flow (40 cm^3^ min^−1^). Finally, to remove the silica template the composite was mixed with the 10% HF solution overnight at room temperature. The obtained product was centrifuged, washed several times with distilled water and ethyl alcohol, and then dried at a temperature of 80 °C overnight. This material is labeled as N,S-MPC. 

#### 2.1.2. Synthesis of Fe-Co/N,S-MPC Catalysts

The series of nanoparticles of iron-cobalt spinel (Fe*_x_*Co_3−*x*_O_4_) covering a wide range of nominal compositions (*x* = 0.5; 1; 1.5; 2; 2.5), was prepared by applying a modified procedure described by Wu et al. [23]. The mixed spinel active phase was in situ deposited on doped mesoporous carbon support (N,S-MPC) using the microwave-assisted hydrothermal route. The corresponding transition metal precursor salts, Co(NO_3_)_2_·6H_2_O and Fe(NO_3_)_3_·9H_2_O, were dissolved in 3.5 cm^3^ distilled water in the appropriate molar ratio and stirred for 30 min. After heating the solution to 80 °C, 1.5 cm^3^ of 25 wt.% ammonia was added and stirred for the 30 min at this temperature. The prepared mixture was next added to a carbon suspension (0.01 g C/1 cm^3^ EtOH), and ultrasonicated for 15 min. The resultant mixture was then transferred to a Teflon lined microwave autoclave (Ertec/MAGNUM II, Wrocław, Poland). The reaction temperature was kept at 150 °C for 30 min. The products were centrifuged and washed several times with distilled water and ethanol, and then dried at a temperature of 80 °C overnight. The obtained final catalysts (iron-cobalt spinel deposited on the N,S-MPC support) is labeled as Fe-Co_*x*/N,S-MPC, where *x* determines the iron content in the sample, e.g., for *x* = 0.5 − Fe-Co_0.5/N,S-MPC. For the sake of comparison cobalt spinel (Co_3_O_4_) deposited on doped mesoporous carbon support was synthesized (denoted Co/N,S-MPC) according to the same procedure described elsewhere. The Co/N,S-MPC catalyst was used as reference material.

### 2.2. Methods

#### 2.2.1. X-ray Powder Diffraction (XRD)

The phase composition of the obtained materials was investigated by means of the X-ray diffraction (XRD) technique. The analysis was carried out using a Mini Flex 600 diffractometer (Rigaku, Tokyo, Japan) equipped with the CuKα electrode (*λ* = 1.540598 Å). Diffractograms were recorded in the 2*θ* range from 5 to 90° with a resolution of 0.02°. The average size of the spinel crystallites was calculated from the half-widths of the recorded peaks (determined using the Fityk software (Wojdyr, Warszawa, Poland) [41]), by applying the Scherrer equation [42].

#### 2.2.2. Raman Spectroscopy (RS)

Raman spectra were recorded at room temperature by an InVia spectrometer (Renishaw, Wotton-under-Edge, UK) equipped with a DMLM confocal microscope (Leica, Wetzlar, Germany) and CCD detector. The lasers with the excitation wavelengths of 514 and 785 nm were used. The measurements were carried out with the resolution of 2 cm^−1^, in the range of 100–1800 cm^−1^. The Fityk software was used to deconvolute Raman spectra of carbons samples [41].

#### 2.2.3. Low-Temperature Nitrogen Sorption

Nitrogen adsorption−desorption isotherms were measured at −196 °C using an Autosorb iQ-MP-AG-AG instrument (Quantachrome, Boynton Beach, FL, USA). Prior to the experiments, each sample was outgassed under a vacuum for 8.2 h at 250 °C. The specific surface areas of the tested materials were determined using the Brunauer-Emmett-Teller (BET) model. Total pore volume (*V*_total_) was determined based on the amount of adsorbed nitrogen at the relative pressure of 0.95. The mesopore volume (*V*_meso_) was calculated using Barrett-Joyner-Halenda (BJH) method while the micropore volume (*V*_micro_) was calculated from the difference between the values of *V*_total_ and *V*_meso_.

#### 2.2.4. X-ray Fluorescence Spectroscopy (XRF) 

The elemental composition of the obtained samples (Fe:Co ratio) was investigated using the X-ray fluorescence spectroscopy (XRF). The measurements were carried out with anARL Quant’X spectrometer (Thermo Fisher Scientific, Waltham, MA, USA), equipped with the commercial analysis UniQuant software.

#### 2.2.5. Thermogravimetric Analysis (TGA)

Thermogravimetric analysis (TGA) measurements were performed by means of a TGA/DSC1 apparatus (Mettler Toledo, Greifensee, Switzerland) equipped with STAR software. The experiments were carried out under air flow (40 mL min^−1^) at the temperature range from 25 to 850 °C with a heating rate of 5 °C min^−1^.

#### 2.2.6. Elemental Analysis 

The elemental composition of the synthesized carbon support was determined by elemental analysis (EA). The measurements were performed on a Micro Cube elemental analyzer (Vario, Langenselbold, Germany).

#### 2.2.7. X-ray Photoelectron Spectroscopy (XPS)

The spectra were collected using a monochromatized aluminum source Al Kα (E = 1486.6 eV). The base pressure in the analytical chamber was 5 × 10^−9^ mbar. The scale of the binding energy value was adjusted to the C 1s reference peak at 284.8 eV. The composition and chemical state of the sample surface were analyzed in terms of areas and binding energies of Co 2p, Fe 2p, O 1s, C 1s, N 1s and S 2p photoelectron peaks. The spectra were fitted using CasaXPS software [43].

#### 2.2.8. Transmission Electron Microscopy (TEM)

Microscopic analysis of the samples was carried out using a Tecnai Osiris microscope (Thermo Fisher Scientific Inc., Carlsband, CA, USA) operating at 200 kV. STEM imaging was performed using a high-angle annular dark-field (HAADF) detector. An analysis of the chemical composition of the samples was performed using an energy dispersive X-ray spectrometer (EDX) equipped with the Esprit microanalysis system (Bruker, Billerica, MA, USA). The STEM images coupled with the EDX elemental mapping were acquired with a sample drift correction using the Bruker Esprit software. Prior to microscopic analysis, the samples were dispersed in ethanol and dropped on a lacey carbon film supported on a copper grid (300 mesh, Agar Scientific, Essex, UK). 

#### 2.2.9. Electrode Preparation and Rotating Ring Disk Electrode (RRDE) Measurements

In order to prepare ink for the electrochemical tests, a mixture of 5.4 mg of the supported spinel catalyst with 62 µL of 5 wt.% Nafion solution, and 938 µL isopropanol was sonicated for 60 min to achieve a good dispersion. The Nafion-to-catalyst mass ratio was equal to 0.33. Then an appropriate amount of the selected ink was pipetted onto the surface of a glassy carbon disc electrode with the resultant loading of the catalysts (spinel and carbon) on the electrode of ~0.2 mg cm^−2^. For comparison, a commercial 20 wt.% Pt on Vulcan XC-72 (Fuel Cell Store, College Station, TX, USA) catalyst was also tested. The ink was prepared according to the same procedure using 10 mg of Pt/C, 33 μL of 5 wt.% Nafion solution, 20 μL of deionized water, and 734 μL of isopropanol. The catalyst loading on the electrode (total mass of the Pt and the C support) was the same as for spinel catalysts.

The activity towards ORR of the obtained catalysts was tested in an alkaline environment (0.1 M KOH, pH = 13) with the use of a PGSTAT 302N potentiostat (Metrohm Autolab B.V., Utrecht, The Netherlands) in a conventional three-electrode cell, equipped with a glassy carbon disk working electrode, concentrically surrounded by a platinum ring (GCE-Pt, surface area GCE = 0.1256 cm^2^), a Pt wire counter electrode, and an Ag/AgCl reference electrode. The electrolyte was saturated by bubbling the corresponding gas (O_2_ or Ar) prior to each experiment, and then during the tests. In order to obtain an electrochemically clean and stable catalyst surface, a modified working electrode was activated by performing several full voltammetric potential cycles (at 100 mV s^−1^) in the potential range from 0 to 1.2 V vs. RHE in a deoxygenated 0.1 M KOH solution, until stable results were obtained. The redox properties of the obtained materials were determined by cyclic voltammetry (CV) in an Ar-saturated electrolyte at a scan rate of 10 mV s^−1^. 

The catalytic activity of the tested materials in the ORR was assessed using linear sweep voltammetry (LSV). Measurements were carried out in O_2_-saturated 0.1 M KOH, at a scan rate of 5 mV s^−1^ on the disc electrode, while the constant potential of 1.2 V vs. RHE was applied to the ring electrode, with several rotating speeds ranging between 800 and 2000 rpm. The applied potentials were iR-corrected and converted to the RHE scale. The registered currents were normalized to the geometric area of the electrode and additionally corrected to the background currents measured in an Ar atmosphere.

## 3. Results

### 3.1. Physicochemical Characterization of a Double-Doped Mesoporous Carbon Support

**Figure 1:**
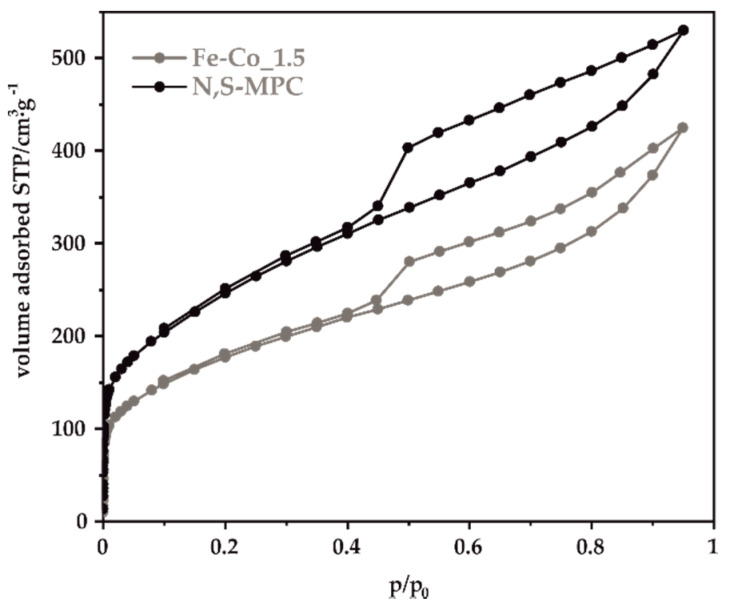
The N_2_-sorption isotherm for the N,S-MPC support and Fe-Co_1.5 samples.

The obtained carbon support was characterized by N_2_-sorption measurements in order to determine its textural parameters. The shape of the obtained nitrogen adsorption-desorption isotherm (Figure 1) exhibits a visible hysteresis loop in the relative pressure range *p*/*p*_0_ = 0.45–0.95 and can be assigned to type IV according to IUPAC classification [44]. The profile of the isotherms at lower relative pressures indicates a small contribution of the micropores.

The calculated values of the BET specific surface area and the pore volumes are listed in Table 1. The obtained N,S-MPC support is characterized by a high specific surface, reaching for bare carbon 728 m^2^·g^−1^, with the predominant contribution of mesopores to the total pore volume.

After the spinel synthesis, the specific surface area decreased by 26–28% except for the sample Fe-Co_1. The decrease in the specific surface area can tentatively be explained by the assumption that spinels nanocrystal plugged a certain amount of pores blocking thereby an access of nitrogen molecules to carbon support pores. Indeed, in the case of Fe-Co_1 extensive agglomeration of the oxides nanocrystals was observed (vide infra), which is in line with such conjecture. These agglomerates reduce contacts of the oxide grains whit the carrier and blocked, therefore, a small fraction of the mesopores which resulted in only a slight reduction of the carbon surface. It is worth noting here that this sample is also outside of general trend of ORR selectivity discussed below.

The amount of the introduced heteroatoms was determined using elemental analysis. The obtained results, indicate the effective incorporation of 2.60% of nitrogen and 1.42% of sulfur into the structure of the synthesized doubly doped carbon (89.6%) material.

**Figure 2:**
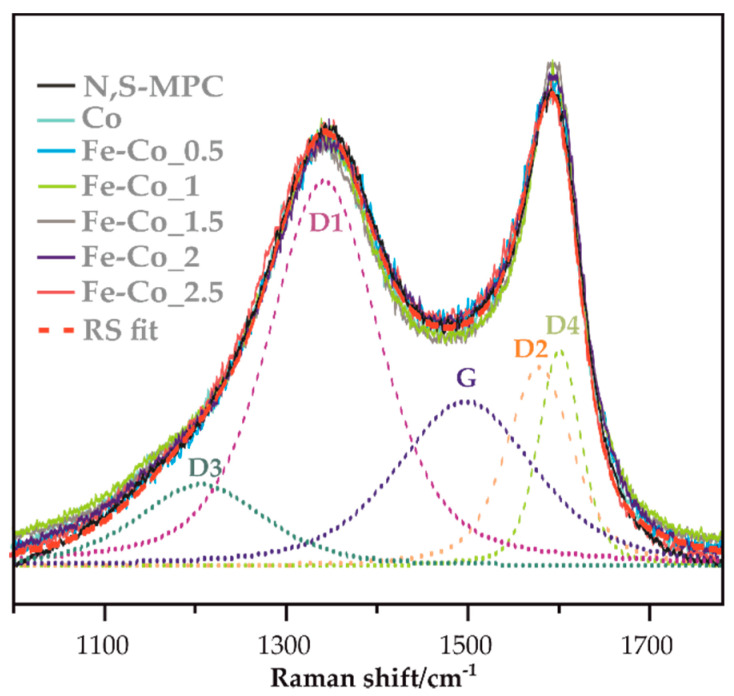
Raman spectra of bare N,S-MPC support and spinel/carbon (indicated in the figure) samples includes deconvolution into individual component signals (dash lines).

In Figure 2 the Raman spectra of bare N,S-MPC support, and for all spinel/carbon samples includes deconvolution into individual component signals (dash lines) are presented [13,45,46]. As can be seen, registered spectra are very similar and the variation of D_3_/G as well as D/G ratios, calculated on the base of performed deconvolution, are not sufficient to draw any conclusions.

### 3.2. Physicochemical Characterization of Catalysts

#### 3.2.1. XRF, TGA, XRD, XPS and Micro-RS Analyses

**Figure 3:**
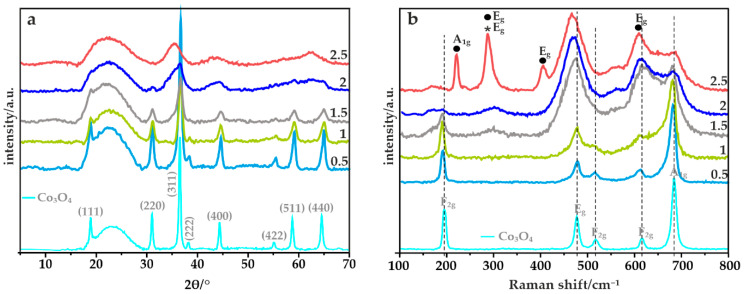
(**a**) XRD patterns and (**b**) Raman spectra of the Fe-Co oxide active phase with different iron content deposited on the N,S-MPC carrier.

The elemental composition of the obtained Fe-Co oxide active phase was determined by the XRF method. The atomic ratios of Fe:Co in the synthesized samples, summarized in Table 2, are highly compliant with the intended values. Moreover, the amount of the oxide phase deposited on the mesoporous carbon matrix was evaluated based on the thermogravimetric analysis, carried out in the flow of air (Table 2). A weight loss of ~70% determined for all the examined catalysts confirms the expected oxide phase loading. The structure and phase composition of the obtained oxide materials deposited on the N,S-MPC carrier were elucidated by means of the X-ray powder diffraction and Raman spectroscopy. The obtained results confirmed the presence of iron-cobalt spinels crystallizing in the cubic *Fd3m* structure (Figure 3a). Furthermore, due to the use of the carbon support, a wide peak at ~23° related to the graphite plane (002) can also be distinguished in all cases [47]. However, depending on Fe*_x_*Co_3−*x*_O_4_ stoichiometry the XRD patterns and RS spectra revealed the presence of additional segregated oxides for *x* > 1.5. The extent of incorporation of the iron to the cobalt spinel structure determines the distribution of the Fe cations into the tetra- and octahedral positions, deciding about the normal or inverse spinel structure. The recorded diffractograms of the samples with *x* varying between 0.5 and 1.5 indicate that a normal iron-cobalt spinel was achieved. Moreover, with the increasing iron content in this range, a noticeable broadening of the diffraction peaks was observed, suggesting that smaller spinel crystallites were obtained.

The Scherrer average size of the spinel nanoparticles changes as 15 nm for Fe-Co_0.5/N,S-MPC > 11 nm for Fe-Co_1/N,S-MPC > 9 nm for Fe-Co_1.5/N,S-MPC. The XRD patterns of the catalysts with higher iron content (*x* ≥ 2) are characterized by a substantial broadening of the reflections and their distinct shift towards lower angles. Such features are compatible with the presence of very small crystallites and/or coexistence of several oxide phases with different compositions, implying segregation of iron and cobalt at high Fe doping levels. However, the observed large broadening of the diffraction peaks prevents reliable and univocal identification of the phase composition of the segregated mixed oxides based on XRD data alone. Further, analysis of this issue was then performed by means of RS and TEM measurements.

Raman spectroscopy, being sensitive to local structure, confirms that upon increasing the iron content the samples with mixed phase composition are obtained (Figure 3b). For instance, in the case of Fe-Co_0.5/N,S-MPC with low Fe content, five bands at 192, 477, 512, 612, and 683 cm^−1^, corresponding to the E_g_, 3xF_2g_, and A_1g_ vibrational modes, confirm the normal iron-cobalt spinel structure [48]. Enhancement of the Fe concentration leads to the appearance of structural changes, manifested by broadening and shifting of the bands towards lower wavenumbers in the Raman spectra [49]. Moreover, for the catalysts where the amount of iron is larger than cobalt additional bands at 223, 289, and 407 cm^−1^ can be distinguished, which were assigned to A_1g_ (Fe_2_O_3_), E_g_ (Fe_2_O_3_, Fe_3_O_4_) and E_g_ (Fe_2_O_3_) vibrational modes. Additionally, it is suggested that the band at ~610 cm^−1^ is a superposition of two bands associated with the presence of both mixed iron-cobalt spinel and Fe_2_O_3_ [50].

**Figure 4:**
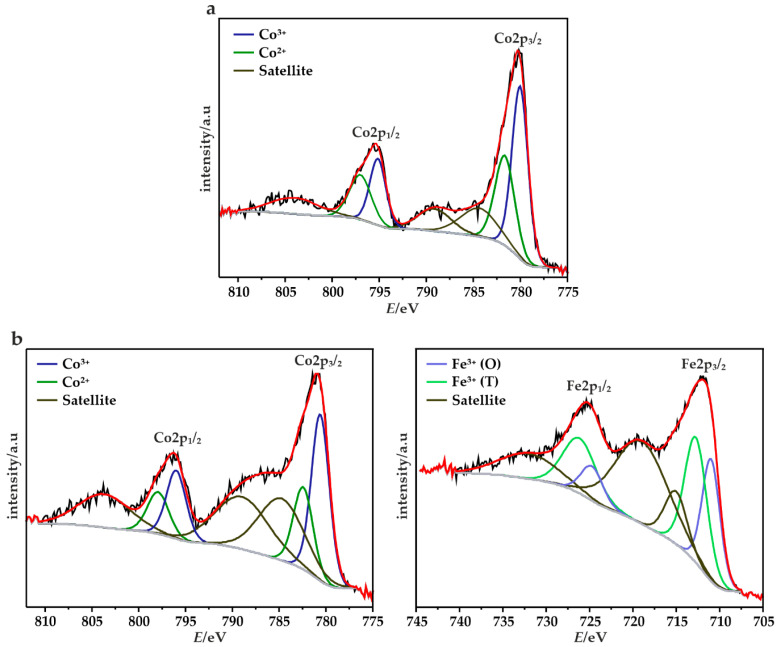
The XPS spectra of the Co_3_O_4_/MPC catalysts in core level spectrum of Co2p (**a**) and of Fe-Co_1.5/MPC catalysts core level spectrum of Co2p and Fe2p (**b**).

In order to obtain the cation oxidation state and the surface chemical composition of the obtained catalysts, X-ray photoelectron spectroscopy (XPS) measurements were conducted. The fitted XPS spectra for the Co 2p and Fe 2p region for the Co_3_O_4_ and Fe-Co_1.5 samples are shown in Figure 4, and calculated chemical composition of the spinels are given in the Table 3. In the case of the mixed iron-cobalt spinel due to the occurrence of an Auger peak from the other respective metal which overlays mainly the 2p_3/2_ part of the spectra the interpretation is not straightforward [51,52]. The spectra of Co 2p show two asymmetric peaks which can be assigned to Co 2p_3/2_ and Co 2p_1/2_ together with corresponding satellite structures.

The deconvolution of these spectra revealed the presence of several overlapping features associated to Co^3+^ (780.1 ± 0.6 and 795.4 ± 0.6 eV) and Co^2+^ (781.7 ± 0.7 and 797.3 ± 0.7 eV) [53]. The observed satellite peaks are typical for spinel structures consists of 3+ cations occupy octahedral lattice sites, filled t_2g_ and empty e_g_ levels, and 2+ cations in tetrahedral sites [54]. However, in the case of Fe-Co samples, the shake-up peaks in the range of 783.9 ± 0.9–788.6 ± 0.5 eV are overlaid due to the Fe LMM line [51]. The XPS spectrum of Fe 2p shows also two peaks corresponding to Fe 2p_3/2_ and Fe 2p_1/2_, which imply the existence of Fe^3+^ cation [25,51,55]. Further deconvolution revealed the contribution of Fe^3+^ in octa- (O) and tetrahedral (T) sites, whereas the visible satellite structures might be evidence for the presence of the small amount of Fe^2+^ in the obtained catalyst [7,55]. In addition, the shake-up peaks in the range 715.1 ± 0.3–719.2 ± 0.2 eV are overlaid by an Auger line coming from the Co [51,52].

Unfortunately, due to the overlapping of the diagnostic 2p and the presence of iron in several forms make quantification not reliable and useful for the establishment of the possible correlation between the content of particular F^2+^/Fe^3+^ contents and catalysts selectivity.

#### 3.2.2. S/TEM/EDX Imaging

**Figure 5:**
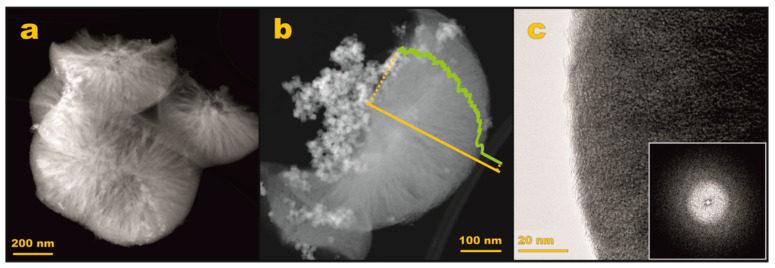
Electron microscopic analysis of the synthesized mesoporous carbon support. HAADF STEM imaging reveals porosity and overall morphology of the support (**a**,**b**), whereas HR TEM (**c**) and calculated FFT pattern (**c**, inset) its amorphous nature.

The shape and morphology of the mesoporous carbon support were examined by transmission electron microscopy, and the obtained results are presented in Figure 5. As can be seen in Figure 5a, the Z-contrast changes along the carbon support grain, indicating variations in their thickness. The character of these variations suggests the presence of microporosity of the examined material. A more detailed insight into the morphology is presented in Figure 3b, where the indicated intensity profile shows that the thickness increases from the edge of the carbon grain towards the center (cf. green line in Figure 5b). Such features along with the rounded shape imply that the vast majority of the carbon support particles exhibit an overall spherulitic shape, characteristic for the operation of a diffusion limited growth mechanism. A high-resolution TEM image (Figure 5c) shows the amorphous (confirmed by FFT analysis, Figure 5c inset) nature of the carbon support grains with the mesopore’s network running preferentially from the center to the edge. 

**Figure 6:**
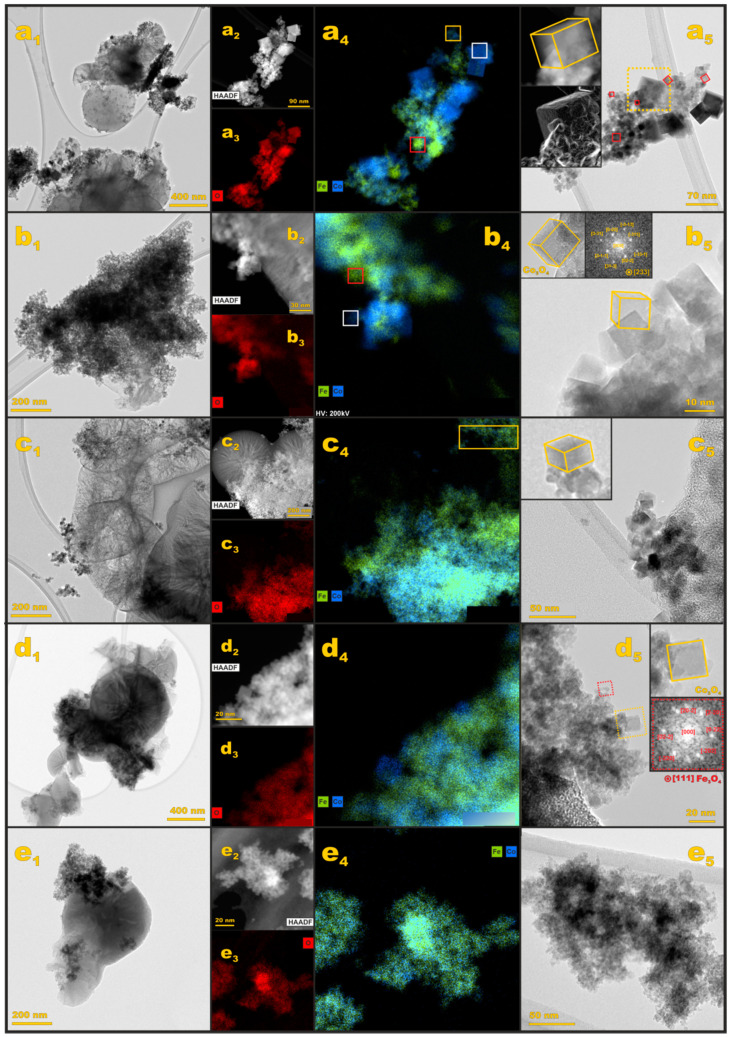
Microscopic TEM analysis of the Fe*_x_*Co_3−*x*_O_4_ active phase with (**a**) *x* = 0.5 (**b**) *x* = 1 (**c**) *x* = 1.5 (**d**) *x* = 2.0 (**e**) *x* = 2.5. First column, (**a_1_**–**e_1_**), presents survey imaging of the samples, EDX results are presented in the **a_2_**–**e_4_** columns, whereas the **a_5_**–**e_5_** column shows magnified TEM images of the Fe-Co oxide nanocrystals.

To elucidate dispersion, elemental composition, and morphology of the oxide active phase a detailed microscopic TEM, HAADF/STEM, and EDX analysis for each iron-cobalt spinel sample were carried out. The results are presented in Figure 6 for the Fe-Co_0.5 (a_1_–a_5_), Fe-Co_1 (b_1_–b_5_), Fe-Co_1.5 (c_1_–c_5_), Fe-Co_2 (d_1_–d_5_), and Fe-Co_2.5 (e_1_–e_5_) samples.

The BF-TEM image of the Fe-Co_0.5/N,S-MPC sample (Figure 6a_1_) shows that the nanometric oxide particles are agglomerated on the carbon support. The EDX analysis (Figure 6a_2_–a_4_) revealed that the nominal Fe:Co = 0.2 ratio is fairly well maintained (cf. yellow square in Figure 6a_4_). Apart from the mixed Fe–Co spinel phase, the EDX mapping confirms also the presence of bare cobalt spinel nanocrystals (Figure 6a_4_, white square) and separated crystalline iron oxides, confirming the Raman results. More sensible shape analysis shows that the bare cobalt and mixed Fe–Co spinel nanocrystals exhibit often a fairly cubic morphology (Figure 6a_5_, yellow and red squares, respectively). In the case of the segregated iron oxide, the observed nanocrystals are very small (*d* < 5 nm) in contrast to the cubic nanocrystals of both spinels. In the latter case the nanocrystals edge length variates from 10 nm for the mixed iron-cobalt spinel to 50 nm for the bare cobalt spinel. 

Strong agglomeration of the active phase was observed for the Fe-Co_1/N,S-MPC sample. The EDX mapping revealed significant segregation of the cobalt and iron-based phases. As it can be inferred from Figure 6b_3_, cobalt spinel nanocrystals (Figure 6b_3_, white square) are surrounded by much smaller nanocrystals containing iron (Figure 6b_3_, red square). The shape analysis confirmed nearly cubic morphology of the cobalt spinel nanocrystals with an average edge length of 10 nm (inset of Figure 6b_5_, a Co_3_O_4_ nanocrystal is viewed along the [233] axis). 

The BF-TEM image of the Fe-Co_1.5/N,S-MPC catalyst shows the lowest extent of the active phase agglomeration, in comparison to other analyzed samples. As it can be deduced from the EDX maps (Figure 6c_2_–c_4_), this sample contains mainly the iron-cobalt spinel nanocrystals, which preserve the nominal ratio of Fe:Co = 1:1 (Figure 6c_4_, yellow square). Nonetheless, a segregated phase of the iron oxides is also present. The iron-cobalt spinel nanocrystals exhibit predominant cubic morphology, with an average edge length of 10 nm.

In the case of the Fe-Co_2/N,S-MPC sample, the active phase is strongly agglomerated on the carbon support. The EDX investigations reveal also some segregation of the cobalt and iron phases (Figure 6d_2_–d_4_). Cobalt-iron spinel nanocrystals exhibit cubic shape with *d* = 10 nm (Figure 6d_5_). Iron-rich nanocrystals have subhedral morphology. The FFT analysis (Figure 6d_5_, inset, red square) indicates the segregated iron oxide phase corresponds to Fe_3_O_4_ spinel structure. 

The BF-TEM imaging of the Fe-Co_2.5/N,S-MPC sample, in turn, revealed strong agglomeration of the active phase. The EDX maps (Figure 6e_2_–e_4_) imply that cobalt and iron are strongly mixed, yet some areas of enhanced cobalt content can be distinguished. The oxide grains are very small (*d* < 5 nm), making the active phase almost amorphous (Figure 6e_5_). 

Summarizing TEM results, a partial segregation of iron oxides was observed in the case of all samples yet for *x* < 1.5 its extent is insignificant, and not detectable by the XRD or RS techniques. Therefore, in the case of Fe_1.5_Co_1.5_O_4_ the active phase may be considered as being practically constituted by the mixed iron-cobalt spinel. It should be emphasized that XRD and RS techniques were not specific enough to distinguish between mixed iron-cobalt spinel and bare cobalt spinel, and only detailed TEM analysis allowed for the proper determination of the phase compositions. 

### 3.3. Electrochemical Reduction of Oxygen 

#### 3.3.1. Cyclic Voltammetry

**Figure 7:**
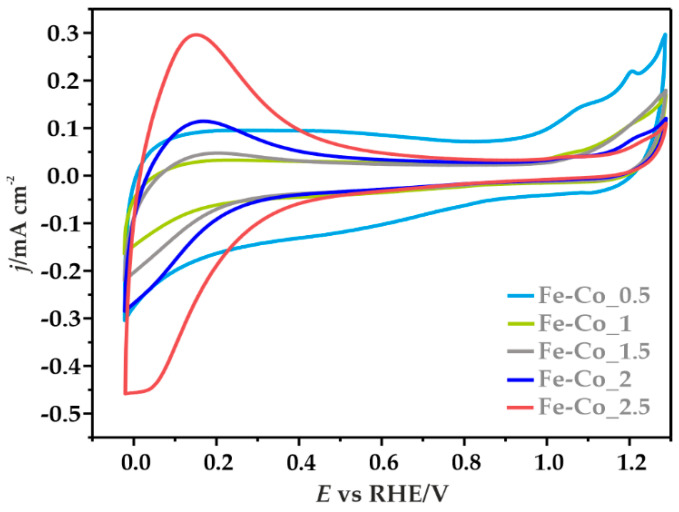
CV profiles for the Fe-Co oxide active phase deposited on the N,S-MPC, recorded in Ar-saturated 0.1 M KOH solution at the scan rate of 10 mV s^−1^.

The redox properties of the obtained catalysts were determined by cyclic voltammetry in an Ar-saturated electrolyte at the scan rate of 10 mV s^−1^ (Figure 7). In the case of Fe-Co_0.5/N,S-MPC two distinct oxidation peaks were recorded at ~1.07 and 1.20 V, along with a slight reduction peak at ~1.13 V, which are related to the following reactions [56]:

3Co^2+^ + 8OH^−^ ↔ Co_3_O_4_ + 4H_2_O + 2e^−^ (1)

Co_3_O_4_ + OH^−^ + H_2_O ↔ 3CoOOH + e^−^ (2)

Some asymmetry of the anode and cathode current density between the visible redox pair, and the absence of a second reduction peak, may suggest *quasi*-reversibility or irreversibility of the involved redox processes [57]. Moreover, in the case of this sample, noticeably higher capacitive currents were recorded, which are consistent with the high contribution of the capacitive effects resulting from the carbon support of high specific surface area [58,59]. Along with the increase in the iron content a systematic increase of the pseudo-capacitive effects on the registered current densities was observed. The well-defined redox peaks at ~0–0.3 V correspond to oxidation/reduction reactions of the iron oxide species [60,61]. The observed peaks were assigned to the reduction of iron oxides and oxyhydroxides to Fe(OH)_2_ and the formation of Fe_3_O_4_ [61]. 

#### 3.3.2. Electrocatalytic ORR Activity 

**Figure 8:**
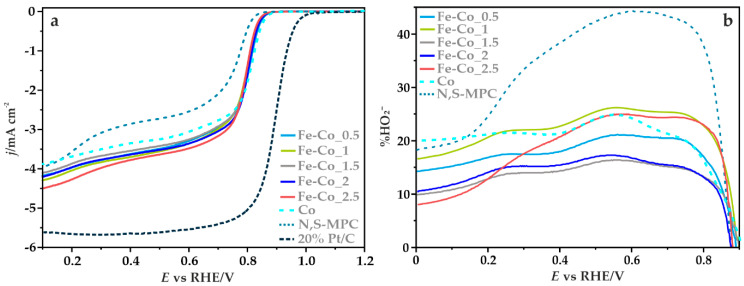
(**a**) The LSV profiles for the Fe-Co oxide active phase deposited on the N,S-MPC support and for the bare carbon support, compared with the spinel Co/N,S-MPC and Pt/C benchmark catalysts recorded in O_2_-saturated 0.1 M KOH solution at 1600 rpm; (**b**) Percentage of ${\mathrm{HO}}_{2}^{-}$ calculated from the RRDE measurements performed in 0.1 M KOH solution at 800 rpm.

The ORR activity and selectivity of iron-cobalt spinel with different iron content, deposited on the N,S-MPC support was determined using the RRDE method. The LSV curves recorded in O_2_-saturated 0.1 M KOH solution with the rotation speed of 1600 rpm are given in Figure 8a. The activity of the examined catalysts and the bare carbon support are compared with the activity of the reference spinel Co/N,S-MPC and commercial 20% Pt/Vulcan XC-72 catalysts.

The doubly doped carbon support exhibits a relatively high onset potential of about 0.83 V, in comparison to the reduction potential registered for bare carbon materials published previously [13]. The onset potential of the ORR (*E_onset_*) was determined as a threshold potential at which the current density of 0.1 mA cm^−2^ is generated in a steady-state RRDE/RDE experiment [62]. The observed improved electrocatalytic activity results from the successful incorporation of nitrogen and sulfur into the mesoporous carbon structure. The two characteristic reduction waves observed for the N,S-MPC support indicate the changes in the oxygen reduction pathway along with decreasing of the potential, which was also confirmed by the parallel analysis using the K-L method. The average number of the transferred electrons (*n*) determined in the range of 0.3–0.5 V is 2.5, which suggests a reduction of oxygen to ${\mathrm{HO}}_{2}^{-}$. However, for lower values of the potential, 0.2 and 0.1 V, higher *n* values were obtained, 3.24 and 3.41, respectively. This observation was also confirmed by the RRDE method (vide infra). Such variation of the *n* values may be associated with the fact that a part of the generated peroxide could further be reduced, increasing the contribution of the stepwise (2e^−^ + 2e^−^) ORR pathway at high overpotentials.

The recorded potentiodynamic curves for the synthetized catalysts are characterized by similar profiles over the entire examined potential window, with the limiting current density of ~4.25 mA cm^−2^. For each sample, the kinetic control region (independent of the rotation rate) takes place at the potentials above 0.8 V, while in the region of higher overpotentials, mixed kinetic–diffusion limitations were observed. The reduction potentials, determined from the voltammetric curves are summarized in Table 4. For the majority of the tested catalysts, they assume the same values (*E*_onset_ = 0.86 V and *E*_1/2_ = 0.78 V). Only for the Fe-Co_2.5/N,S-MPC sample a slight decrease in the onset potential was observed. The catalytic activity (assessed based on the reduction potential) in ORR of the supported Fe-Co spinel nanoparticles is significantly higher, in comparison to the corresponding values for the doubly promoted carbon support alone, however, they are similar to the akin spinel Co/N,S-MPC catalyst. These results are comparable to the behavior of similar electrocatalysts based on the transition metal oxides published previously [13,24].

To delineate the pathways of the oxygen reduction reaction on the obtained catalysts, from the recorded RRDE profiles the number of the transferred electrons and the percentage of the produced ${\mathrm{HO}}_{2}^{-}$ species with respect to the total ORR products were calculated according to Equations (3) and (4), respectively:

(3) $$n=\frac{4\times I_{d}}{I_{d}+\frac{I_{r}}{N}}$$

(4) $${\%\mathrm{HO}}_{2}^{-}=\frac{200\times \frac{I_{r}}{N}}{I_{d}+\frac{I_{r}}{N}}$$

where *I_d_* is the current measured at the GC disk, *I_r_* is the current measured at the Pt ring, and *N* is the collection efficiency (*N* = 0.249).

Figure 8b presents the percentage content of ${\mathrm{HO}}_{2}^{-}$ oxidized on the ring electrode as a function of the potential applied to the disc electrode. Production of peroxide on the studied catalysts begins at the potential ~0.85 V, then it rises sharply until reaching the maximum value at around ~0.55 V. The highest amount of the intermediate product at this potential value is the result of the use of the doubly promoted carbon support, which in an alkaline environment is able to reduce oxygen mainly through a two-electron pathway, leading to the formation of peroxide intermediates [63,64]. The average percentage of the formed ${\mathrm{HO}}_{2}^{-}$ (in the range of 0.1–0.5 V) is about 32% for N,S-MPC, whereas for the Fe-Co catalysts it varies from 14% to 22%. A very similar value of ~22% was observed for the reference cobalt spinel electrocatalyst supported on the same N,S-MPC carrier. As the applied potential decreased further, a decline in the production of hydrogen peroxide was observed, which indicates an increased contribution of the four-electron reaction pathway. The lowest amount of the formed intermediate products in the almost entire potential range, and thus the highest number of transferred electrons, was registered for the catalyst at *x* = 1.5. 

In the potential range of 0.1–0.5 V, the average number of electrons (determined by the RRDE method) changed in the following order: 3.73 for Fe-Co_1.5 > 3.71 for Fe-Co_2 > 3.66 for Fe-Co_2.5 > 3.65 for Fe-Co_0.5 > 3.56 for Fe-Co_1, whereas for the N,S-MPC support, and the Co/N,S-MPC catalyst it was equal to 3.36 and 3.57, respectively. Despite that for the catalyst with *x* = 2.5 the maximum on the profile representing the formation of ${\mathrm{HO}}_{2}^{-}$ versus potential is relatively high (Figure 8b), a quite large average number of the transferred electrons was noted. This is due to the fact that after reaching the maximum, a much faster decrease in the production of ${\mathrm{HO}}_{2}^{-}$ was observed for the Fe-Co_2.5 catalyst in comparison to the other samples. It suggests a further reduction of the generated ${\mathrm{HO}}_{2}^{-}$ species, leading to an ensuing increase in the contribution of the sequential ORR pathway (2e^−^ + 2e^−^) as the potential decreases.

## 4. Discussion

**Figure 9:**
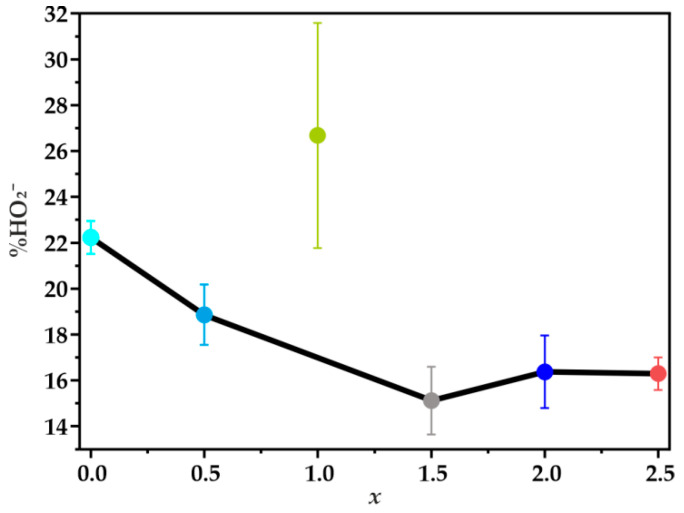
The average percentage of the ${\mathrm{HO}}_{2}^{-}$ in the range of 0.1–0.5 V, calculated from the RRDE measurements performed in 0.1 M KOH solution at 800 rpm as a function of the iron content in the Fe*_x_*Co_3−*x*_O_4_ spinel catalyst dispersed on the N,S-MPC support.

Based on the performed experiments, it was shown that Fe-Co oxide active phase dispersed on doped mesoporous carbon support is a fairly active catalyst for oxygen reduction reaction in an alkaline environment. The presented results indicate a significant role of the composition, aggregation, and segregation of the Fe-Co oxide active phase on the selectivity in ORR. It was found that in addition to the desired mixed iron-cobalt spinel phase in the synthesized samples several segregated oxide phases were present, such as Fe_2_O_3_, Fe_3_O_4,_ and Co_3_O_4_ spinel. Although the XRD results indicate the presence of different phases only for the samples with *x* ≥ 2, the more detailed analysis by transmission electron microscopy showed that phase segregation at variable extent occurred practically in the entire composition range, during the commonly applied hydrothermal synthesis. The identified individual phases and their degree of agglomeration on the carbon support directly influence the selectivity of the obtained catalysts. The selectivity of the catalyst was significantly improved when part of cobalt cations was substituted by iron, and a single phase mixed spinels were formed. The lowest content of the produced peroxide species, and thus the highest number of transferred electrons, was obtained for the Fe-Co_1.5 sample with the maximum amount of mixed Fe-Co spinel phase (Figure 9). The TEM/EDX imaging showed a much lower degree of agglomeration of the nanocrystals deposited on the support as compared to the other tested materials in this sample. Furthermore, the predominant content of the beneficial mixed spinel phase with the nominal Fe:Co = 1:1 ratio (denoted Fe-Co_1.5/N,S-MPC) was confirmed based on the EDX studies.

The highest amount of the generated hydrogen peroxide, and thus the lowest number of the transferred electrons obtained for the Fe-Co_1 catalysts (comparable to that of the bare Co spinel) can be explained by TEM results, which reviled strong agglomeration of the oxide phase. Additionally the EDX analysis of the catalyst with *x* = 1 revealed that in this case the dominant phase was cobalt spinel surrounded by iron oxides, whereas the preferred iron-cobalt spinel phase was scarcely present. The production of hydrogen peroxide for both Fe-Co_1 and Co samples was very similar (21.77 versus 21.51%), therefore it might be concluded that in both samples the ORR reaction mostly takes place on cobalt spinel component. Since no mixed spinel phase appeared in this sample the HO_2_^−^% is shown as a separate point in Figure 9. When the number of the introduced iron increases, *x* = 2, and *x* = 2.5, the generation of hydrogen peroxide is enhanced again due to the high content of the segregated iron oxide phases, particularly for the Fe-Co_2.5 sample. The presence of iron oxides, which was detected by the Raman spectra and confirmed by TEM analysis, apparently promote the ${\mathrm{HO}}_{2}^{-}$ production. 

## 5. Conclusions

The obtained results indicate that bare carbon support doped with N and S show relatively high activity in the ORR in the alkaline environment. However, its selectivity towards the 4e^−^ the process is low, as evidenced by the very high production of hydrogen peroxide. Single phase mixed Fe-Co_1.5/N,S-MPC catalyst (with the Fe:Co composition 1:1) achieved the best selectivity outperforming the reference Co_3_O_4_/N,S-MPC sample. It clearly indicates that well defined mixed iron-cobalt spinel allows for the deep 4e^−^ reaction, whereas the highly dispersed (nearly amorphous) phase of iron oxides prefer the 2e^−^ pathway. Dispersion of the Fe-Co_1.5 phase on N,S-doped carbon support contributed to significant improvement in the selectivity and activity of the catalyst, as documented by the decrease in ${\mathrm{HO}}_{2}^{-}$ production and increase of the *E*_onset_ potential. Additionally, the results indicate a significant influence of the dispersion and composition of the active phase on the selectivity of the developed catalysts and a destructive role of spurious iron phase for the catalyst selectivity. 

## Figures and Tables

**Table 1:** Textural parameters of the carbon support for the bare N,S-MPC carrier, and spinel/N,S-MPC samples.

Sample	Textural Parameters			
	*S_BET_*/m^2^ g^−1^	*V_total_*/cm^3^ g^−1^	*V_micro_*/cm^3^ g^−1^	*V_meso_*/cm^3^ g^−1^
N,S-MPC	728	0.822	0.055	0.767
Fe-Co_0.5	531	0.591	0.063	0.528
Fe-Co_1	691	0.802	0.084	0.718
Fe-Co_1.5	524	0.658	0.049	0.609
Fe-Co_2	523	0.678	0.131	0.547
Fe-Co_2.5	533	0.639	0.067	0.572

**Table 2:** The average size of crystallites, total loading, and elemental composition of the Fe-Co oxide nanoparticles deposited on the N,S-MPC support.

Sample	*D*_Scherrer_/nm	Elemental Composition/at.% ^1^		Oxide Phase Loading on Carbon Support/wt.% ^2^
		Fe	Co	
Fe-Co_0.5	15	16	84	24
Fe-Co_1	11	32	68	31
Fe-Co_1.5	9	47	53	31
Fe-Co_2	n.d.	63	37	28
Fe-Co_2.5	n.d.	80	20	28

^1^ XRF; ^2^ TGA; n.d.—not determined.

**Table 3:** Binding energy (E, eV) of the representative XPS emissions for the investigated spinels and calculated chemical composition.

*E*/eV			Chemical Composition/at.%					
			Co	Fe-Co_0.5	Fe-Co_1	Fe-Co_1.5	Fe-Co_2	Fe-Co_2.5
296.0–281.5	C 1s		83.27	71.57	65.28	62.3	64.06	66.34
408.3–394.9	N 1s		3.45	2.06	2.42	2.42	2.16	2.71
172.4–161.2	S 2p		1.47	1.05	1.35	1.43	1.33	1.23
537.0–527.5	O 1s		8.82	15.86	19.98	20.85	20.84	19.33
**Co 2p**								
780.1 ± 0.6	Co^3+^	2p_3/2_	0.91	0.97	1.21	1.26	0.7	0.19
781.7 ± 0.7	Co^2+^		0.58	0.88	0.89	0.67	0.53	0.42
783.9 ± 0.9	Satellite-1 /Fe LMM		0.34	1.27	1.26	1.16	1.13	0.96
788.6 ± 0.5	Satellite-2 /Fe LMM		0.25	0.87	0.82	1.17	1.03	0.92
795.4 ± 0.6	Co^3+^	2p_1/2_	0.35	0.45	0.53	0.55	0.26	0.06
797.3 ± 0.7	Co^2+^		0.31	0.46	0.57	0.37	0.26	0.2
803.5 ± 0.6	Satellite-3		0.25	0.68	0.45	0.71	0.44	0.26
			2.99	5.58	5.73	5.89	4.35	3.01
**Fe 2p**								
710.5 ± 0.4	Fe^3+^ (O)	2p_3/2_		0.24	0.72	1.07	1.05	1.18
712.4 ± 0.4	Fe^3+^ (T)			0.98	1.20	1.37	1.58	1.43
715.1 ± 0.3	Satellite-1 /Co LMM			0.89	0.9	0.57	0.69	0.65
719.2 ± 0.2	Satellite-2 /Co LMM			1.03	1.13	2.12	1.62	1.83
724.2 ± 0.6	Fe^3+^ (O)	2p_1/2_		0.13	0.29	0.37	0.64	0.51
726.0 ± 0.4	Fe^3+^ (T)			0.44	0.6	0.89	0.88	0.9
731.7 ± 0.5	Satellite-3			0.17	0.4	0.71	0.82	0.88
			0	3.88	5.24	7.10	7.28	7.38

**Table 4:** Electrocatalytic properties of the Fe-Co oxides active phase deposited on the N,S-MPC support, compared with the spinel Co/N,S-MPC. The average number of the transferred electrons and the formed ${\mathrm{HO}}_{2}^{-}$ intermediates were determined by the RRDE method, in the potential range of 0.1–0.5 V.

Sample	*E*_onset_/V	*E*_1/2_/V	*n*	$HO_{2}^{-}/\%$
Co	0.87	0.79	3.56 ± 0.01	22.24 ± 0.72
Fe-Co_0.5	0.86	0.78	3.63 ± 0.02	18.86 ± 1.32
Fe-Co_1	0.86	0.78	3.47 ± 0.09	26.68 ± 4.90
Fe-Co_1.5	0.86	0.78	3.70 ± 0.03	15.12 ± 1.48
Fe-Co_2	0.86	0.78	3.67 ± 0.03	16.38 ± 1.58
Fe-Co_2.5	0.85	0.78	3.67 ± 0.01	16.30 ± 0.71

## Data Availability

The data presented in this study are available on request from the corresponding author.
